# Future trends in the supply and demand for radiation oncology physicists

**DOI:** 10.1120/jacmp.v11i2.3005

**Published:** 2010-04-12

**Authors:** Michael D. Mills, Judah Thornewill, Robert J. Esterhay

**Affiliations:** ^1^ Department of Radiation Oncology University of Louisville School of Medicine Louisville KY USA; ^2^ Department of Health Management and Systems Sciences University of Louisville School of Public Health and System Sciences Louisville KY USA

**Keywords:** residency program, CAMPEP, demand, supply, manpower, radiation oncology physicists, medical physicists

## Abstract

Significant controversy surrounds the 2012 / 2014 decision announced by the Trustees of the American Board of Radiology (ABR) in October of 2007. According to the ABR, only medical physicists who are graduates of a Commission on Accreditation of Medical Physics Education Programs, Inc. (CAMPEP) accredited academic or residency program will be admitted for examination in the years 2012 and 2013. Only graduates of a CAMPEP accredited residency program will be admitted for examination beginning in the year 2014. An essential question facing the radiation oncology physics community is an estimation of supply and demand for medical physicists through the year 2020. To that end, a Demand & Supply dynamic model was created using STELLA software. Inputs into the model include: a) projected new cancer incidence and prevalence 1990–2020; b) AAPM member ages and retirement projections 1990–2020; c) number of ABR physics diplomates 1990–2009; d) number of patients per Qualified Medical Physicist from Abt Reports I (1995), II (2002) and III (2008); e) non‐CAMPEP physicists trained 1990–2009 and projected through 2014; f) CAMPEP physicists trained 1993–2008 and projected through 2014; and g) working Qualified Medical Physicists in radiation oncology in the United States (1990–2007). The model indicates that the number of qualified medical physicists working in radiation oncology required to meet demand in 2020 will be 150–175 per year. Because there is some elasticity in the workforce, a portion of the work effort might be assumed by practicing medical physicists. However, the minimum number of new radiation oncology physicists (ROPs) required for the health of the profession is estimated to be 125 per year in 2020. The radiation oncology physics community should plan to build residency programs to support these numbers for the future of the profession.

PACS numbers: 87.90.+y, 87.53.‐j

## I. INTRODUCTION

It is generally accepted that all professions need to plan for the future, and radiation oncology physics is no exception to this tenet. A key aspect of such planning is to project future supply and demand from existing statistics, data and modeling. The 2012/2014 initiative is bringing a sense of rapid change to our profession. The AAPM Board of Directors met at the RSNA in November of 2008 to finalize a contract with the New York Center for Health Workforce Studies of the University at Albany (State University of New York). The purpose of the contract is to assess the current complexity of medical physics with respect to our training programs and pathway to board certification.

The Medical Physicist Workforce Subcommittee will be directing the Workforce Survey. It will be important for all stakeholders to be recognized and all impact factors to be evaluated and included in the final scope of work. The Workforce Survey may reveal challenges and changes within the profession consequent to transformation in regulatory, technological and reimbursement patterns. It will be important to evaluate all of these factors in order to predict workforce dynamics, and provide for the future education and training of medical physicists. A reasonably accurate projection of supply and demand positions the profession for the future in several respects. If too many medical physicists are trained, the resulting oversupply could lead to unemployment and lower wages. If too few training positions are available, the resulting undersupply could result in extended working hours and a lower quality of life. As a significant level of energy is required to create and maintain Commission on Accreditation of Medical Physics Education Programs, Inc. (CAMPEP) accredited training programs, it is perceived that the immediate danger is the likelihood of creating too few training positions rather than too many. Presently fewer than 30% of medical physicists undergo a formal residency program after graduation from an academic program.

Radiation Oncology Physicists (ROPs) face three major issues:

- The percent rate of retirement of ROPs between the years 2010 and 2020 is approximately triple the rate that existed between 1990 and 2000 (see discussion below).
- Cancer incidence is projected to grow at approximately 2% per year. This will result in a projected increase in the number of cancer patients of approximately 30% between the years 2000 and 2020.
- In 2014, ROPs must graduate from a CAMPEP accredited residency program in order to enter the certification process of the American Board of Radiology.^(1)^

Beginning in 2014, only a graduate of a CAMPEP residency program wishing to enter radiation oncology physics will be able to:

a) sit for certification in Therapeutic Radiological Physics by the American Board of Radiology,
b) be considered a qualified medical physicist (QMP) as defined by the American Association of Physicists in Medicine and the American College of Medical Physics, and
c) receive a license to practice medical physics in states that require licensure.

Since American Board of Radiology (ABR) Certification is required for entry into the profession, by the year 2014 the medical physics profession must create enough residency programs and training positions to meet the demand. The Task Group‐133 Report suggests this need might be met in part through the creation of affiliated residency programs administrated in association with CAMPEP accredited residency programs.^(2)^ Software to manage such affiliated programs is available for use by the medical physics community.^(3)^ It is generally anticipated that the number of programs and training positions must grow by a factor of between five and eight in order to meet demand but, as of this writing, there has been no rigorous demand and supply model to defend this assertion.

## II. MATERIALS AND METHODS

A Demand & Supply model assumes that factors affecting both supply and demand may be either positive or negative. An increasing incidence of cancer would increase demand for radiation oncology physics services, while greater efficiencies in radiation therapy delivery might decrease demand. Having more residency programs would increase the supply of medical physicists, while increasing retirement rates might decrease the supply. It is therefore important to identify and quantify as many factors as possible that influence both the demand and supply of medical physicists. (See Table 1 and Table 2 for the scope of input information required for the model.)

**Table 1:** Iinput information required for the model.

*Year*	*New Cancer Patient Prevalence x 10^6^ – average annual rate of 1.0192^(7)^*	*ABR Physics Diplomats in Therapeutic Radiological Physics*	*Number AAPM Full Members Only*	*Number Estimated to be Practicing Full Time in Rad. Onc. (at 80% of previous column)*	*Clinical FTE – number assuming an average 0.75 clinical effort of previous column*	*Number in Rad. Onc. Retiring by Year (see text for complete model)*	*Assumed Number of Patients per FTE Physicist (Abt Studies I,II,III)^(4,5,6)^*	*Number of Therapy Residents Entering CAMPEP Programs*
1990	1.00	50	1949	1559	1169	22	424	
1991	1.01	61	2005	1604	1203	22	424	
1992	1.02	67	2166	1733	1300	24	424	
1993	1.04	49	2299	1840	1379	26	424	
1994	1.06	64	2280	1824	1368	26	424	
1995	1.08	93	2442	1954	1465	27	424	
1996	1.10	73	2573	2058	1544	29	424	
1997	1.12	108	2610	2088	1566	29	407	1
1998	1.15	76	2668	2134	1601	30	391	4
1999	1.17	64	2749	2199	1650	31	374	7
2000	1.20	65	2804	2243	1682	31	358	10
2001	1.22	70	2906	2325	1744	33	341	13
2002	1.25	95	3441	2753	2065	39	325	16
2003	1.28	86	3527	2822	2116	40	321	19
2004	1.30	71	3657	2926	2194	41	317	22
2005	1.33	109	3865	3092	2319	43	313	25
2006	1.36	121	4100	3280	2460	46	309	
2007	1.38	116	4545	3636	2727	51	304	
2008	1.41					57	304	
2009	1.44					59	304	
2010	1.47					65	304	
2011	1.50					68	304	
2012	1.53					73	304	
2013	1.56					79	304	
2014	1.59					87	304	
2015	1.62					91	304	
2016	1.65					92	304	
2017	1.68					95	304	
2018	1.71					96	304	
2019	1.74					102	304	
2020	1.77					106	304	

Notes: Col. 3 – information courtesy of Stephen Thomas, American Board of Radiology; Col. 4, 5, 6, 7 – information courtesy of Michael Woodward, American Association of Physicists in Medicine; Col. 9 – numbers not known precisely; are estimated by the authors based on the number of CAMPEP programs existing each year and on CAMPEP records for more recent years.

**Table 2:** Age profile of AAPM members (as of January 2008).

*Age*	*# Full Members*	*Age*	*# Full Members*
97	1	62	94
93	3	61	109
91	2	60	108
90	3	59	120
89	3	58	114
88	4	57	119
87	4	56	115
86	7	55	124
85	5	54	129
84	8	53	153
83	9	52	144
82	7	51	143
81	7	50	141
80	10	49	143
79	8	48	153
78	11	47	168
77	8	46	181
76	13	45	181
75	10	44	187
74	22	43	179
73	36	42	185
72	25	41	142
71	29	40	174
70	41	39	156
69	41	38	157
68	52	37	148
67	59	36	135
66	88	35	123
65	78	34	131
64	80	33	159
63	65	32	125

Source – Michael Woodward, American Association of Physicists in Medicine.

The Demand information used as input to the model is:

- Cancer incidence and prevalence 1990–2020 (American Cancer Society)^(4)^
- Median new cancer patients treated per Clinical FTE Radiation Oncology Physicist (Abt Study of Medical Physicist Work Values for Radiation Oncology Physics Services)^(5)^ ^,^ ^(6)^ ^,^ ^(7)^

The Supply information used as input to the model is:

- Non‐CAMPEP ROPs trained and ABR Certified 1990–2009 and projected through 2014 (calculated by the authors based on information provided by Stephen Thomas, American Board of Radiology, and Michael Woodward, American Association of Physicists in Medicine)
- CAMPEP Residency ROPs trained and ABR Certified 2000–2009 and projected through 2020 (calculated by the authors based on information provided by Stephen Thomas, American Board of Radiology, and Michael Woodward, American Association of Physicists in Medicine)
- Number of Working ROPs 1990–2008 (calculated by the authors and based on information provided by Michael Woodward, American Association of Physicists in Medicine)

The demand forecasts are based on the forecasts of incident and prevalent cancer cases, as well as the estimated age and gender‐specific oncologist visit‐rates for cancer patients developed by the National Cancer Institute (NCI) in July 2006.^(4)^ While constructing the model, several assumptions were made that affect demand and supply, both positive and negative. These are listed below:

Positive Demand Assumptions:

- One half of all new cancer patients are treated with radiation therapy.^(8)^
- The median number of new cancer patients managed by an FTE ROP was measured as 424 in 1995, 325 in 2002 and 304 in 2007.^(5)^ ^,^ ^(6)^ ^,^ ^(7)^ This factor has a most significant impact on the model. In the absence of additional data, it was assumed that there was a linear decline in the number of patients managed by ROPs between 1995 and 2007. The median number was assumed to be 424 before 1995 and 304 after 2007 (see Table 1, column 8).
- A working ROP is assumed to spend three‐quarters of the time on clinical service and therefore to provide 0.75 clinical FTE. The remainder of time is allocated to teaching, research, administration, and other nonclinical services such as serving on institutional committees, radiation safety, and JCAHO facility support. Therefore the ratio of working ROPs to Clinical FTEs is 1.3333. This number varies widely, depending on the type of practice and nature of the institution. The assumption in this article is based on a limited survey of various practice types and on information contained within the Abt reports.^(5)^ ^,^ ^(6)^ ^,^ ^(7)^

Negative Demand Assumptions:

- 80% of Full Members of the American Association of Physicists in Medicine are involved in radiation oncology physics service. Of all ABR Certified Diplomates between 1990 and 2007, the totals were 1438 (69%) in Therapeutic Radiological Physics, 504 (24%) in Diagnostic Radiological Physics, and 149 (7%) in Medical Nuclear Physics. It was recognized that many of the diagnostic and medical nuclear certificates are owned by those also certified in therapy physics and who are primarily practicing therapy physics. Therefore, the 80% practicing therapy physics relationship from the professional practice survey is assumed to hold for this analysis (based on information provided by Stephen Thomas, American Board of Radiology).
- Since most ROPs retire between ages 65 and 69, then for a given year, an average of the number of AAPM members between 65 and 69 is used. It was assumed that this percentage remained constant between 1990 and 2007. Assuming the AAPM membership has remained relatively stable, it is possible to use the information in Table 2 to project the number of physicists retiring as a fraction of the entire membership. When performed, this calculation results in an average retirement rate of 0.014 for the years between 1990 and 2007. (Since this era is before the baby boomers begin to retire, a constant rate is not unreasonable.) For years 2008–2020, this same method was used, assuming a rolling five‐year average age based on the age profile of the current AAPM membership. For example, for 2010, the number of medical physicists between 63 and 67 (as of 2008) was taken, and averaged over five years, to determine the number of retiring medical physicists in 2010. This number was multiplied by 0.8 to reflect the number of Therapy ROPs retiring and again by 0.75 to determine the number of retiring therapy clinical FTEs (calculated by the authors and based on information provided by Michael Woodward, American Association of Physicists in Medicine).

Positive and Negative Supply Assumptions:

- Assume that figures supplied by the American Board of Radiology and the American Association of Physicists in Medicine accurately reflect the number of CAMPEP and non‐CAMPEP trained and certified physicists entering the workforce between 1990 and 2007.
- Assume a linear ramping of CAMPEP Residency graduates between 2008 and 2020. Evaluate the consequences of training between 100 and 175 residents per year in CAMPEP programs in 2020.

The Demand & Supply workforce model was designed and programmed using the STELLA modeling application (ISEE Systems, Inc., Lebanon NH). The model features a means to vary the rate of retirement from that initially predicted by the model and to vary the number of expected CAMPEP graduates in the year 2020. Additional output information includes the number of work hours per week of the median ROP, and the cost of maintaining training programs with respect to the number of physicists trained. The Demand & Supply model for ROPs consists of five components: 1) cancer demand, 2) ROP demand, 3) ROP supply, 4) training funds, and 5) operating funds. The model conveys information and passes data between components to produce the information desired.

Training and Operating Funds Model:

These models contain the following assumptions:

- The inflation rate will rise at 3% per year for training wages 1987–2020.
- The annual cost to support a resident for a year (salary plus benefits) was estimated at $32,000 in 1987. This is equivalent to approximately $60,000 in 2008.
- The salary plus benefit compensation for the median physicist in 1987 was $90,000. This along with a 4% annual increase yields salary and benefit figures experienced today by AAPM membership.
- Annual training funds needed in millions are the dollar amounts to support salaries and benefits of all residents in all United States CAMPEP programs.
- Annual faculty salary and benefit funds to support residency are the dollar amounts to support faculty salary and benefits for all programs in the United States, assuming each resident requires a total 0.25 FTE.

We designed a test of the model using a subset of the data. The presented dataset contains as input the number of physicists successfully passing the oral ABR examination in Therapeutic Radiological Physics between 1990 and 2009. We compare these numbers in this model with projections from the model but missing four years of this data: 2006–2009. The purpose of this test is to see if the model would predict over the five‐year period after 2005 an appropriate Demand and Supply profile for the profession.

## III. RESULTS & DISCUSSION

**Figure 1:**
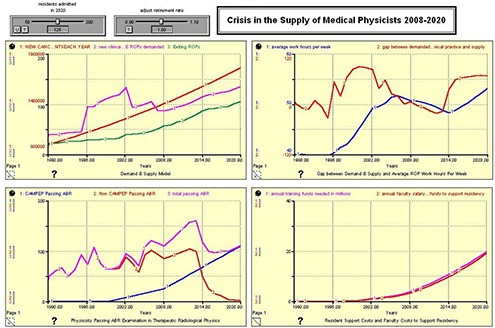
ABR 125 CAMPEP – Accredited Residency Positions Available in 2020. The presented dataset does not contain as input the number of physicists successfully passing the oral ABR examination in Therapeutic Radiological Physics between 2006 and 2009.

Respecting the test described above, the assumptions were run for 125 ROPs entering CAMPEP residency training in 2020. The results from the model missing the ABR oral examination data 2006–2009 are reported in Fig. 1, and should be compared with results from the model containing that data (Fig. 3). Here, the discussion is limited to red line in the graph in the upper right quadrant – the gap between demand for Working ROPs in Clinical Practice and supply. What we determined is that, while the model performed properly and as expected, the run without the 2006–2009 data underestimated the number of new ROPs passing the ABR Board Examination in Therapeutic Radiological Physics in 2008 and 2009 by approximately 25–30% – or approximately 100–120 ROPs. Since we knew and properly projected the numbers of CAMPEP residency graduates over these years, the additional numbers of physicists would seem to represent non‐CAMPEP trainees that 1) were attracted into the field because of the shortage and salary scale, and 2) have expedited their training, perhaps because of the 2012–2014 mandate. The model demonstrates the numbers of ABR certified ROPs are currently exceeding demand and creating the beginning of an oversupply. Although in 2009 supply approximately equals demand, between 2010 and 2014 the oversupply may reach 50–60 ROPs per year. The model predicts the oversupply is expected to reverse once the 2012/2014 mandate takes effect. The current job market bears out this observation, as some new ROPs are finding it difficult to locate employment.

**Figure 2:**
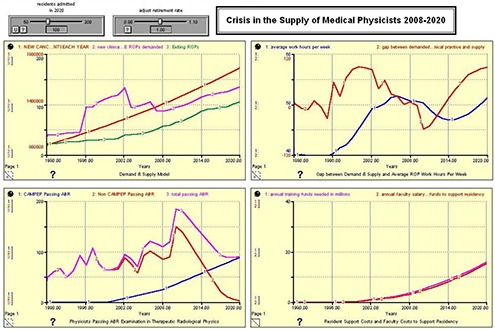
100 CAMPEP – Accredited Residency Positions Available in 2020.

**Figure 3:**
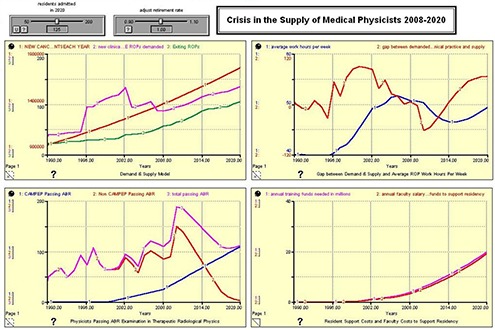
125 CAMPEP – Accredited Residency Positions Available in 2020.

**Figure 4:**
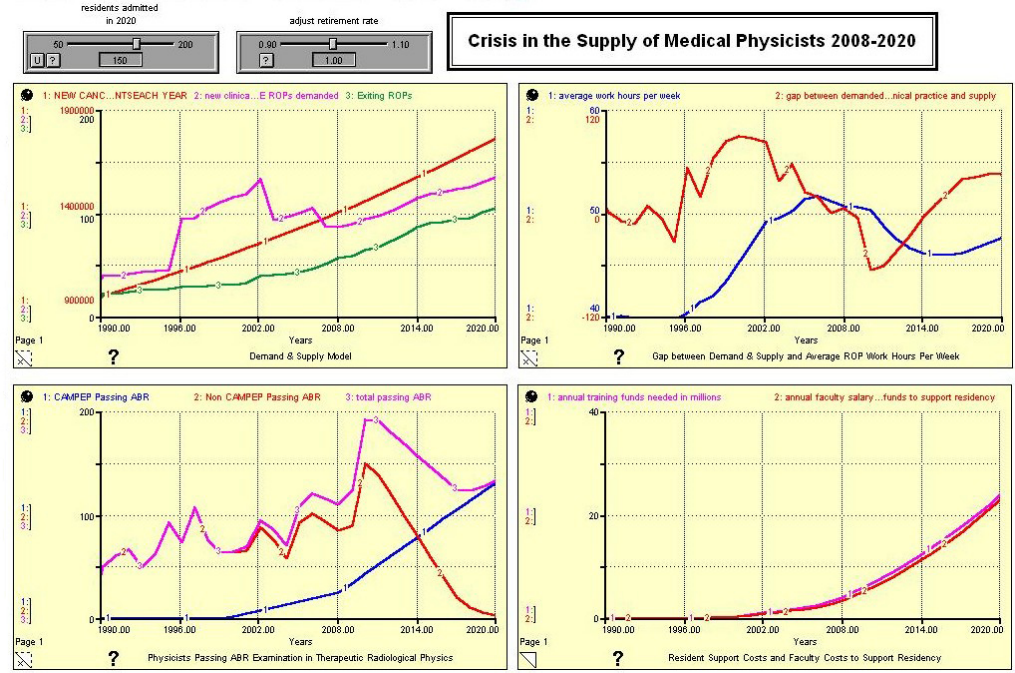
150 CAMPEP – Accredited Residency Positions Available in 2020.

**Figure 5:**
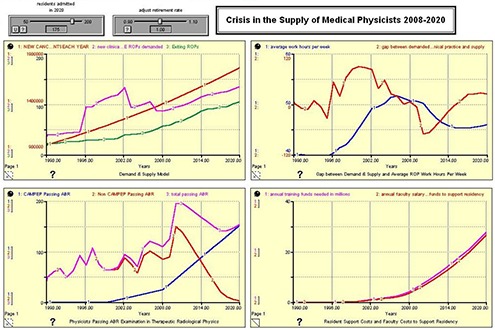
175 CAMPEP – Accredited Residency Positions Available in 2020.

Figures 2, 3, 4, and 5 show the results of the model run for an anticipated 100, 125, 150, and 175 residents admitted to United States CAMPEP programs in the year 2020. The results show the effect on the gap between demand and supply, the median ROP work week, and program costs. Each figure has four quadrants, and each quadrant is discussed as follows:

**Legend for all Figures**

**Upper left quadrant**: (Red) New Cancer Patients Each Year; (Pink) New Clinical FTE ROPs demanded; (Green) Exiting ROPs. **Lower left quadrant**: (Blue) CAMPEP <trainees> Passing ABR; (Red) Non‐CAMPEP <trainees> Passing ABR; (Pink) Total <trainees> Passing ABR. **Upper right quadrant**: (Blue) Average Work Hours per Week; (Red) Gap between Demanded Working ROPs in Clinical Practice and Supply. **Lower right quadrant**: (Pink) Annual Training Funds Needed in Millions; (Red) Annual Faculty Salary and Benefit Funds to Support Residency.

**Demand and Supply Model**. The red line shows the anticipated number of newly diagnosed cancer patients each year between 1990 and 2020. The green line shows the anticipated number of ROPs that exit the profession each year due to retirement from the field. The pink line shows the number of ROPs demanded based on demographics and the median number of new patients for which a physicist is responsible. Note the large increase centered on the year 2002. This may possibly be due to the emergence of a number of physics‐intensive special procedures (IMRT, IGRT, prostate seed brachytherapy, stereotactic procedures, etc.) in the general radiation oncology community, and the willingness of the payment system to support these emerging technologies. Since the retirement rate is held constant for this analysis, the Demand & Supply Model graph does not change between Fig. 1 and 4.

**Physicists Passing ABR Examination in Therapeutic Radiological Physics**. The model assumes a linear increase in the number of residents entering CAMPEP residency programs between 2007 and 2020. Additionally, the model assumes a drop in the number of physicists who are able to enter the profession after 2012 without going through a CAMPEP residency program. It is assumed that, although this pathway remains open in 2012 and 2013 for graduates of CAMPEP academic programs, the number will already be diminishing by 2014 due to the elimination of the non‐CAMPEP pathway, the declining job market for entry‐level medical physicists without residency experience, and the availability of CAMPEP residency positions for graduates of CAMPEP academic programs. Note that the total number passing the ABR examination lags the number entering residency training by three years.

**Gap Between Demand & Supply and Average ROP Work Hours per Week**. This quadrant illustrates the gap between demand and supply and the work hours per week of ROPs between the years 1990 and 2020. The work hours per week are determined based on the manpower available to treat the estimated number of cancer patients treated with radiation oncology. It is important to note that the model assumes an ROP will be able to provide services for 304 patients per year as reported in the Abt III survey between the years 2007 and 2020. It is certainly possible that ROP work respecting time‐consuming special procedures will become more efficient. If that is indeed the case, the presented results might well overestimate medical physicist demand by 2020. Note the large gap between supply and demand in the early 2000s. This gap is ascribed to the increasing number of special procedures in radiation oncology as mentioned earlier. Consequently, the median ROP is able to handle fewer patients annually. The results suggest that creating only 100 CAMPEP residency training positions would be damaging to the profession. Demand is seen to exceed supply and work hours increase beyond reasonable limits. Creating 125 positions is the minimum that would seem to allow for stability in the profession, given work stress and overtime demands involved at this level. Providing the community with 150 training positions would marginally meet anticipated demand, while 175 training positions would actually approach equivalence between supply and demand. It should be noted that the pool of qualified ROPs is relatively robust (approximately 2500–2700 in the United States at present) and that there is some elasticity in the community to take up the slack if fewer than 150 CAMPEP residency training positions are created. However, if too few positions are created by 2020, there is the possibility in the succeeding years that more medical physicists will retire or leave the profession, with a potential negative impact on patient care.

The model is sensitive respecting several factors, but demonstrates elasticity respecting others. One sensitive factor is the median number of patients an ROP supports on an annual basis, as supplied from the Abt data. ROPs could become more efficient in performing special procedures and able to handle more patients per year by 2020. Conversely, ROPs might be faced with new technologies that allow them to handle fewer patients per year. Since these factors are difficult to predict, it is assumed in this model that the median ROP will manage 304 new patients per year through 2020. However, a 10–20% change in this number has a significant effect on the model. For example, using 125 certified physicists in 2020 as an endpoint, if the median ROP practice is 360 patients per year in 2020, the work week of the ROP is decreased from over 55 hours per week to 45 hours per week; there is also a decrease in the gap between demanded and supplied physicists of about 20%. On the other hand, using 125 certified physicists supplied in 2020 and assuming the median ROP will manage 280 patients per year, the work week is over 65 hours per week, with a 40% increase in the gap between supply and demand. Clearly, changes in this median patient workload figure on the order of that measured in the Abt study over the past 14 years will have a significant effect on the validity of the model.

The retirement of ROPs is another important factor; however, it is one that demonstrates elasticity. A 10% change in the retirement rate decreases or increases the median workweek by about 5 hours per week, with a 15% decrease or increase in the gap between demanded and supplied ROPs. Respecting manpower, there seems to be a lot of elasticity in the system, as the pool of clinical FTEs available to do physics work is expected to expand or contract based on need and incentives. Retired physicists may be persuaded to work part time if the rewards are sufficient.

There is also a potential major impact of the current economic crisis on the delay of ROP retirement. This is expected to manifest beginning in 2009, and may continue for between five and ten years. This factor, combined with the anticipated oversupply over the next five years, may negatively impact the profession and we may see hiring and/or salary freezes before the nation's economy and the supply/demand for ROPs recover.

One factor that is difficult to predict is the number of ROPs currently training in non‐CAMPEP programs that have expedited the progress of their training in order to enter the certification pathway before the 2012 deadline. If these numbers approach 200 ABR certified ROPs per year between 2009 and 2012, there could be a sharp oversupply of ROPs by up to 100 per year through the year 2014. This oversupply could negatively affect the profession's ability to place residents, as well as maintain the current entry level salaries.

**Resident Support Costs and Faculty Costs to Support Residency**. It is assumed that the FTE faculty/staff ROP support for a residency program is 0.25 FTE per resident. The salary plus benefits for a resident are also estimated at 0.25 that of a faculty physicist. Therefore the cost of providing support for a resident during training is approximately the same in dollars as the level of faculty support to mentor and train the resident.

Today, we train approximately 100 physicists per year in working positions and about 25 per year in CAMPEP residencies in the United States.

- 100 physicists×$130,000 in salary &benefits=$13,000,000 (working positions)
- 25 physicists×$60,000 salary &benefits=$1,500,000 (CAMPEP residency positions)
  If all these physicists were trained in CAMPEP residency programs:
- 125 physicists×$60,000 salary &benefits=$7,500,000 (CAMPEP residency positions)

There is therefore already more than enough money in the system to train medical physicists. The community should therefore redirect the funding to pay for medical physicists in CAMPEP residency positions rather than in work positions. Affiliated or distributed residency programs are potential mechanisms to accomplish this objective.

Despite these advantages, the current economic climate might negatively impact the willingness to allocate funds for medical physics training. It might also affect hospital funding decisions with regard to major radiation oncology equipment purchases. These decisions would have an effect on the quality of training available, as well as the number of training slots offered. These factors are difficult to predict and beyond the scope of this model.

## IV. CONCLUSIONS

It is interesting to conjecture to what degree the parameters to be studied by the AAPM and the New York Center for Health Workforce Studies might validate the predictions of this STELLA model. Results from this Workforce Study are expected toward the end of 2009. The Task Group‐133 report stated, “There is evidence that 200 to 400 qualified clinical medical physicists are required to join the workforce annually (various surveys).”^(2)^ Medical physicists faced an unexpected and dramatic demand for ROP services in the early part of the decade. Also, the community is faced with the 2012/2014 mandate concerning ABR certification. It is easy in the face of these experiences and facts to be unreasonably pessimistic respecting the ability to create a sufficient number of residency positions to meet the demand. However, the demand is not anticipated to be as great as estimated by Task Group‐133; the true demand is likely to be around 150 per year. The large increase in demand for ROP services centered around 2002 is unlikely to be repeated unless a new technology requiring significant ROP manpower emerges. Sufficient funding already exists in the system to provide for the mentoring and training of physics residents; however, such funding needs to be redirected into CAMPEP accredited residency programs or affiliated programs. As more residency programs are accredited and as affiliated programs emerge, the number of training positions may reasonably be expected to grow to meet the demand required by 2020.
